# Dihydrofolate Reductase Gene Variations in Susceptibility to Disease and Treatment Outcomes

**DOI:** 10.2174/138920210793360925

**Published:** 2010-12

**Authors:** Bahram S. Askari, Maja Krajinovic

**Affiliations:** 1 Research Center, CHU Sainte-Justine, Montreal, QC, Canada; 2 Department of Pediatrics, University of Montreal, Canada; 3 Department of Pharmacology, University of Montreal, Canada

**Keywords:** Gene, dihydrofolate reductase (DHFR), polymorphisms, disease susceptibility, methotrexate (MTX), therapeutic response.

## Abstract

Dihydrofolate reductase (DHFR) catalyzes the reduction of dihydrofolate to tetrahydrofolate (THF). THF is needed for the action of folate-dependent enzymes and is thus essential for DNA synthesis and methylation. The importance of this reaction is demonstrated by the effectiveness of antifolate medications used to treat cancer by inhibiting DHFR, thereby depleting THF and slowing DNA synthesis and cell proliferation. Due to the pivotal role that DHFR plays in folate metabolism and cancer treatment, changes in the level of DHFR expression can affect susceptibility to a variety of diseases dependent on folate status such as spina bifida and cancer. Likewise, variability in DHFR expression can affect sensitivity to anti-cancer drugs such as the folate antagonist methotrexate. Alterations in DHFR expression can be due to polymorphisms in the DHFR gene. Several variations have recently been described in DHFR, including promoter polymorphisms, the 19-bp deletion allele and variations in 3’UTR. These polymorphisms seem to be functional, affecting mRNA levels through various interesting mechanisms, including regulation through RNA interference. Several groups have assessed the association of these polymorphisms with folate levels, risk of cancer and spina bifida as well as the outcome of diseases treated with MTX. The latter may lead to different treatment schedules, improving treatment efficacy and/or allowing for a reduction in drug side effects. This review will summarize present knowledge regarding the predictive potential of DHFR polymorphisms in disease and treatment.

## INTRODUCTION

Dihydrofolate reductase (DHFR) is a member of the reductase enzyme family, which is ubiquitously expressed in all organisms. At the transcriptional level, DHFR is governed by a TATA-less promoter that is controlled by numerous transcription factors, including Sp1 and E2F that are important for its regulation throughout the cell cycle [1, 2]. Levels of this enzyme peak at the G1/S cell cycle boundary. Autoregulation, through DHFR-RNA interactions, has also been reported [3, 4]. DHFR catalyzes the NADPH-dependent reduction of dihydrofolate (DHF) to tetrahydrofolate (THF) needed for several one-carbon transfer reactions in purine and pyrimidine synthesis [2]. DHFR is also needed for the intracellular conversion of synthetic folic acid, consumed in supplements and fortified foods, into the THF forms that can participate in folate/homocysteine metabolism. Reduction of DHFR enzymatic activity diminishes the THF pool inside the cell affecting the level of folate coenzymes and thus purine and pyrimidine synthesis [1]. This may as well influence homocysteine levels and methylation processes, since methyl-THF is needed for the remethylation of homocysteine to methionine, thereby ensuring the provision of S-adenosylmethionine (SAM) necessary for most biological methylation reactions [5]. DHFR inhibition is essential to the action of antifolate medications used to treat cancer and some inflammatory diseases [6-9]. 

The *DHFR* gene family includes the functional *DHFR* gene and four other intronless pseudogenes (*DHFRP1–4*) [10]. The functional gene is located at chromosome 5q11.2-13.2 and is expressed in three mRNA isoforms with alternatively spliced 3’UTR ends [11]. The changes in DHFR expression or activity can be partly due to the functional polymorphisms in the *DHFR* gene, thereby influencing a risk of folate-dependent diseases. Importantly, gene variations may also affect therapeutic responses to antifolates, leading to lower treatment efficacy or higher adverse drug event frequency. This review will summarize several studies that analyzed whether *DHFR* gene polymorphisms may affect disease susceptibility or antifolate treatment outcomes.

## *DHFR* POLYMORPHISMS AND DISEASE SUSCEPTIBILITY

Neural tube defects (NTD) are a group of common birth defects with a prevalence of approximately 1 per 1,000 in Europe [12]. It is well documented that low serum folate and high homocysteine levels are associated with an increased risk of NTD, explaining the preventive effect of folic acid supplement [13, 14]. Change in activity of the folate cycle enzymes may affect the folate levels and affect NTD development. It is, therefore, not surprising that polymorphisms of folate-dependent enzymes of both mother and child, such as methylene tetrahydrofolate reductase (*MTHFR*), have been shown to contribute to the risk of NTD [15, 16]. Neural tube closure occurs during a period of rapid cellular proliferation and DHFR activity may be a crucial factor in maintaining optimal DNA synthesis during this time [13]. The most extensively studied *DHFR* polymorphism is a *19bp *insertion to deletion in the first intron that has been evaluated as a risk factor for NTD in several studies (Table **1**). Johnson *et al*., 2004 [17], suggested that the deletion *(del)* allele could affect gene expression, since the Sp1 transcription factor binding site is located within the deleted sequence. The first, a small case-control study, provided evidence that the risk of having a child with spina bifida is higher for women with the *DHFR 19bp* *del/del* genotype than it is for those with the remaining genotypes [17]. The same authors [18] reported a higher risk of pre-term delivery in patients with the *del* allele. Van der Linden *et al*., 2007 [19], reported an absence of association between *del/del* genotype and spina bifida risk, whereas Parle-McDermott *et al*., 2007 [13], observed a lower risk of having a child with NTD in women with the *del* allele. It has also been reported that individuals with the *del/del* genotype have lower homocysteine [20] and increased serum and red blood cell folate levels [14]. A non-significant increase in mRNA levels for homozygous *del* individuals [13], and subsequently a significant increase in DHFR expression with the number of *del* allele [21], were reported. This would support the possible maternal protective role of the 19-bp *del *allele in NTD by increasing the amount of DHFR available to reduce DHF to THF. However, others reported an absence of association between del allele and mRNA levels [19] or even suggested in the recent cross-sectional, population-based study [22] that the *del/del* genotype resulted in a diminished capacity of the enzyme to reduce folic acid.

It seems that the role of *DHFR* polymorphisms in NTD risk still remains to be determined, since results regarding *19bp del* allele are inconclusive. The abrogation of the Sp1 binding site by the deletion allele or its close location to the splice donor site (60bp) may suggest a functional role of this polymorphism, yet contradictory results have been obtained. The other polymorphisms that are in linkage disequilibrium (LD) with *19bp* insertion to deletion variation may be responsible for the observed results or discrepancies. Indeed, the high extent of LD was noted in the *DHFR* gene with *19bp* insertion to deletion being in LD with the promoter polymorphisms [23]. The same polymorphism is shared among several haplotypes, showing the necessity of estimating the impact based on the analysis of both individual polymorphisms and haplotypes. In some instances the haplotypes may provide more precise information for predicting disease risk than individual polymorphisms [24]. 

DHFR is an important folate cycle enzyme required for nucleic acid synthesis as well as homocysteine remethylation, suggesting that *DHFR* polymorphisms may play a role in cancer susceptibility as well. It is possible that similarly to *MTHFR*, the *DHFR* gene variations may play a dual role. Polymorphisms associated with a higher expression of DHFR may protect against cancer, due to the higher levels of 5,10-methylene-THF needed for thymydylate synthesis, whereas a change in the 5-methyl-THF pool may affect methylation reactions and, consequently, increase cancer risk. Both genomic DNA hypomethylation and gene-specific promoter CpG island hypermethylation are important epigenetic mechanisms of carcinogenesis [5, 25]. Indeed, a protective role of the *DHFR* *19 bp* *del* allele in adult acute lymphoblastic leukemia (ALL) patients has been reported [26], which was further potentiated when the *del *allele was combined with the *TT677* genotype of *MTHFR*, previously shown in several studies to reduce the risk of ALL [27, 28]. 

Analysis of the *DHFR 19-bp *insertion to deletion polymorphism in relation to breast cancer susceptibility did not reveal a significant association with overall breast cancer risk [21, 29]. However, when analyses were performed following stratification according to multivitamin use [21], an association appeared significant in patients that used the multivitamin supplements. Individuals with the *del/del* genotype had a 50% increase in breast cancer risk compared with individuals without this genotype [21]. Although several cohort studies suggested that higher folate intake was associated with lower breast cancer risk, a recent large screening trial showed that higher supplement intake may increase the risk of breast cancer in postmenopausal women [30]. The authors [30] further hypothesize that, because of the complexity of folate function, it is possible that both deficiency and abundance of folate may contribute to breast carcinogenesis at different stages of tumor development or in different tumor phenotypes. 

It is thus possible that genetic variations along with dietary intake of folate and methionine influence cellular one-carbon metabolism and methyl-donor status, which may also affect susceptibility to other cancers such as colorectal carcinoma. Recent studies have shown the potential relationship between germline variants in methyl-group metabolism genes and promoter CpG island methylation in colorectal tumors [31], which seems to be a specific molecular pathway for colon carcinogenesis. Curtin *et al*. [32] looked into an association among genetic polymorphisms relevant to folatemediated one-carbon metabolism, including *DHFR* *19-bp* insertion to deletion polymorphism and colon cancer risk, but did not find any association with the *del* allele [32]. In contrast, a large population-based study that analyzed 395 tag SNPs (a sufficient number of polymorphisms to define common haplotypes, as based on LD) in 15 folate-pathway genes identified two tag SNPs in the *DHFR* gene (intronic *rs1677693* A to C and *rs1643659* A to G polymorphisms) associated with lower colorectal cancer risk [33]. The analyzed population was on a folate fortified diet, but the observed protective effect was particularly obvious in non–multivitamin supplement users, confirming previous observations that the effect of folate cycle gene variants may be potentiated or abrogated by dietary folate intake. 

## *DHFR* GENE POLYMORPHISMS AND RESPONSE TO TREATMENT

The same gene variations that favor THF accumulation may both protect against cancer establishment (as shown in ALL patients [26]) and affect antifolate treatment being responsible for individual differences in terms of survival. This paradoxical dual and opposite effect was recently described as ‘‘false-friend allele’’ behavior [26], and has also been previously noted for *MTHFR* variant alleles [34]. Higher DHFR activity and THF accumulation can contradict a cytotoxic effect of antifolates, thereby reducing treatment efficacy. Methotrexate (MTX) is an important antifolate widely used in the treatment of several malignancies, including acute lymphocytic leukemia, non-Hodgkin's lymphoma, osteosarcoma and choriocarcinoma [35]. DHFR, as a major MTX target, plays an important role in the development of MTX resistance in ALL. In both experimental and clinical settings, altered levels of DHFR and/DHFR gene amplification were found in relapsed leukemia patients and in leukemia and colon cell lines manifesting MTX-resistant phenotypes [8, 36-38]. Of note is that several mechanisms may contribute to the development of MTX resistance. This review is, nevertheless, limited only to *DHFR* gene variations and other mechanisms are reviewed in detail elsewhere [9]. 

Regarding the potential role of *DHFR* polymorphisms in response to MTX treatment (Table **1**), Goto *et al*., 2001 [39], described one of the first variations in *DFHR* while analyzing the 3′-untranslated region (UTR) of the human *DHFR* gene transcript. They discovered *C829T* substitution located 223 base pairs downstream from the stop codon and positioned between the first and second polyadenylation site. This polymorphism seems to influence DHFR expression, which increased with the number of *T* alleles being highest in *TT* individuals [39]. Consequently, the *C829T*-associated increase in DHFR expression may reduce MTX-related cytotoxicity. Indeed, reduction in sensitivity to MTX driven by this polymorphism was subsequently documented by Mishra *et al*., 2007 [40], who further elucidated the functional role of this variation*.* They showed that this SNP is located near the microRNA (miR)-24 3' UTR binding site and that it affects DHFR expression by interfering with miR-24 function. Analysis *in vitro* using Chinese hamster ovary derived cell lines that lack DHFR showed that the cells with the *T* allele bind miR-24 less efficiently, resulting in a 2-fold increase in DHFR mRNA half-life and, hence, higher DHFR mRNA and protein levels. This in turn affected sensitivity to MTX. The cells with the *T* allele were 4-fold more resistant to MTX as compared with cells without this allele [40]. It is known that miRNAs play an important role in different biological processes, such as cell proliferation, cell death, stress resistance and fat metabolism through the regulation of gene expression [41]. They may also be differentially expressed in human cancers or may act as oncogenes and tumor suppressors by targeting key regulators of cell growth [42, 43]. Moreover, translational control mediated by miRNAs plays an important role in the mechanism of cellular resistance to anti-cancer drug treatment [44]. The work by Mishra *et al*. [40, 45, 46] was the first to show that genetic polymorphisms may affect drug sensitivity acting through this mechanism. Several groups reported that *C829T* is non-polymorphic (or appears with very low minor allele frequency) in Caucasians [13, 20]. Nevertheless, it would be worth verifying whether this is also true at the transcriptional level and does not result from the difficulty in designing the primers for DNA amplification (i.e., pseudogenes and repetitive elements). 

Changes in the level of DHFR expression and consequently in sensitivity to MTX can be also due to the genetic polymorphisms located in the promoter. The polymorphisms in the 2 kb region upstream of the first or minor transcription initiation site (corresponding to the minor *DHFR* promoter) were recently analyzed in a cohort of childhood ALL patients [23]. Association of individual polymorphisms and resulting haplotypes with ALL outcome revealed that a reduction in relapse-free survival was associated with *A* and *C* alleles located at positions 317 and 1610 upstream from a minor transcription initiation site, respectively. The association was also noted with the haplotype harboring these alleles, arbitrarily named haplotype *1. Haplotype *1 conferred higher transcriptional activity, as shown by reporter gene assay and quantitative mRNA analysis, likely explaining a worse prognosis in patients carrying this haplotype. In addition to the minor promoter, the human *DHFR* gene also has a downstream major promoter [3, 4]. This adjacent regulatory region is located between minor and major transcript initiation sites and has been shown to act as a non-coding interfering transcript, controlling the transcription of productive mRNA from the major promoter [4]. The polymorphisms of this region were recently defined [24] and tagging variations were subsequently analyzed in the same group of ALL patients [24]. The haplotype analysis revealed diversification of haplotype *1 [23] into five subtypes, and only one of those, *1b, was responsible for the lower relapse-free survival observed in ALL patients, defining more precisely the relapse predisposing variations of *DHFR*. This association seems to be confined to patients with high-risk of relapse, as defined by classical clinical prognostic criteria, and it was further validated in an additional cohort of ALL patients. The *1b subtype was characterized by a particular allele combination defined by allele *T* and *A* at positions 35 and 308 from the first transcription initiation site, respectively, compound length polymorphisms composed of *9bp* insertion at position 63 and triple *9bp* element at position 91. The *9bp* repeat element of the compound length polymorphism was initially observed by Fujii *et al*. [47] and resembles that described in *DHFR 5’UTR* [19] and a mismatch repair gene (*hMSH*3) overlapping *DHFR *[48], although the sequence alignment and the number of repeats were to a certain extent differently resolved. Importantly, *1b was the only haplotype *1 subtype associated with higher mRNA levels and was predicted in silico to affect the structure of the major promoter [24]. It has been shown [3, 4] that the DHFR repression is regulated by a non-coding interfering transcript and that this regulation is due to the formation of a stable complex between non-coding RNA, which also acquires a different conformation, and the major promoter. It is, thus, possible that the *1b haplotype could affect the function of the non-coding transcript, resulting in an observed increase in mRNA levels and a higher risk of ALL relapse [24].

Low-dose MTX is considered the ‘gold standard’ of therapy for rheumatoid arthritis (RA) patients [49]. Nevertheless, there is considerable inter-individual variation in its clinical efficacy. A substantial number of patients do not respond to treatment, whereas others (10-30%) develop drug side effects requiring discontinuation of therapy [50, 51]. Wessels *et al*., 2006 [50], analyzed the association of genetic polymorphisms of the folate pathway with MTX efficacy, expressed as a disease activity score, and methotrexate toxicity, specifically respiratory, gastrointestinal, skin, mucosal and hepatic adverse drug events (Table **1**). Among *DHFR* polymorphisms, they analyzed *DHFR G-473A (rs1650697)* replacement in *5’ UTR* and *DHFR A35289G (rs1232027)* substitution positioned relative to the translation initiation site. No association was found, whereas in contrast, Chandran *et al*. [52] found an association of the *A* allele of *A35289G* polymorphism with MTX efficacy in patients with psoriatic arthritis**. **The study conducted in RA patients from northern India [53] addressed additional *DHFR A/T* polymorphism in *3’UTR* (position 1171 of mRNA, *rs7387*) and revealed its contribution to MTX efficacy only in a multivariate model when analyzed together with other variations of folate-dependent enzymes. Regarding the potential functional role of *DHFR* polymorphisms selected for rheumatoid and psoriatic arthritis study, little can be said. The *G-473A* variation in *5’UTR* (rs1650697) corresponds to *C35T* substitution (given relative to the forward strand and first transcription initiation site) described in ALL patient analysis [24]. Individually this polymorphism does not seem to have an impact on mRNA levels. However the *T* allele is one of the alleles of haplotype *1b, which, as described above [24], seems to increase DHFR expression. The polymorphism in *3’ UTR* such as *rs7387* [53] may potentially affect mRNA stability. However, it is not clear what the functional role [54] of the *A35289G* variation located several Kb downstream from the 3’UTR would be (based on dbSNP data [55]). Another polymorphism in LD with this *DHFR* variation may possibly explain the positive association reported.

## CONCLUSION

DHFR is a critical folate cycle enzyme targeted by antifolate medication used in the treatment of cancer and rheumatoid arthritis. The change in DHFR expression and activity caused by genetic polymorphisms may affect an individual’s predisposition to respond to the treatment in terms of efficacy and drug side effects. Due to the crucial role DHFR plays in the conversion of DHF to THF required for nucleic acid synthesis and methylation reaction, *DHFR *gene polymorphisms might affect diseases dependent on folate status, such as cancer and spina bifida. The genotype-phenotype relationships have only begun to unravel. The results obtained hold promise for the future. They are still scarce and sometimes contradictory requiring further analysis and replication. It is clear that other polymorphisms of the folate pathway, dietary intake and different genetic and epigenetic mechanisms beyond genetic polymorphisms may contribute toward the variability in treatment responses, the understanding of which would allow such information to be used in disease prevention and treatment tailored to individuals.

## Figures and Tables

**Table 1:** Summary of the Studies Associating *DHFR* Polymorphisms with Disease Susceptibility or Response to Treatment

Location/Position	Polymorphism**	Impact	Related Disorders	Reference

Intron 1	19-bp insertion /deletion	Low-serum folate/ high homocysteine, change in mRNA levels	NTD Breast cancer	[13, 17] [21]
Intron 3	A10372C *(rs1677693)* A8890G *(rs1643659)*	unknown	Colon cancer	[33]
3'-UTR	C829T	Interfering with miR-24 function, higher DHFR mRNA and protein levels	MTX resistance	[40]
Minor promoter*	C-1610G or T (rs1650694) A-317/G *(rs408626)*			[23]
Major promoter*	G308A *(rs1105525)* C35T *(rs1650697)* Length polymorphism 63/91: 9-bp insertion deletion/ 9-bp repeat *(rs3045983/ - )*	Higher DHFR expression	Higher risk of relapse in ALL	[24]
Downstream to 3' UTR	A35289G *(rs1232027)*	Unknown	MTX efficacy in patients with psoriatic arthritis	[52]
3'UTR	A1171T *(rs7387)*	Unknown	MTX efficacy in patients with RA	[53]

Table summarizes positive associations described in the text. Details of other analysis which did not necessarily reveal significant associations are given in the text. DHFR, dihydrofolate reductase; MTX, methotrexate; ALL, acute lymphoblastic leukemia; RA, rheumatoid arthritis; NTD, neural tube defects; del, deletion.

* polymorphism in the *DHFR* minor and major promoter defining *1 and *1b haplotype, respectively associated with higher DHFR expression and higher risk of relapse in ALL patients. Position of the polymorphism is given relative to the transcription or translation initiation site, or refers to the position within indicated intron.

** rs SNP number from dbSNP database at National Center for Biotechnology Information (NCBI) is provided as long it is available.
